# Having cake and eating too: The benefits of an intermediate larval form in a brittle star *Amphiodia* sp. opaque (Ophiuroidea)

**DOI:** 10.1002/ece3.10298

**Published:** 2023-07-17

**Authors:** Nicole N. Nakata, Richard B. Emlet

**Affiliations:** ^1^ Oregon Institute of Marine Biology, University of Oregon Charleston Oregon USA

**Keywords:** development, facultative feeding, larva, larval nutrition strategies, ophiuroid

## Abstract

Most marine invertebrate larvae either obligately feed or depend on maternally provided reserves during planktonic development. A small number of species have the capacity to do both, in a mode of development known as facultative planktotrophy. We describe facultative feeding in a larva from the Oregon coast, and identify it as being an undescribed species in the genus *Amphiodia*, which we refer to as *Amphiodia* sp. opaque. We quantified the effects of food on larval and juvenile quality by culturing larvae, collected as embryos, with and without microalgal food at 15°C. The resulting juveniles were monitored under conditions of starvation. A cohort of juveniles of larvae caught as plankton was subjected to the same starvation treatment for comparison with our laboratory‐reared larvae. We observed benefits to offspring that received food: larvae provided with microalgae developed more quickly and metamorphosed at higher rates. Furthermore, juveniles resulting from fed larvae were larger and were able to avoid starvation for longer after metamorphosis. Our results varied across two experimental years, suggesting that provisions provided by parents vary between populations and years. Juveniles from planktonic larvae exhibited sizes not statistically different from larvae cultured in the absence of food, but died from starvation more quickly.

## Graphical abstract

We describe an unusual brittle star larva that represents an intermediate evolutionary stage between feeding and nonfeeding larvae. Larvae of this species are capable of feeding, but can forego food and make a juvenile. Offspring that ate as larvae had shortened development times, higher percent metamorphosis, larger size, and longer juvenile survival times.

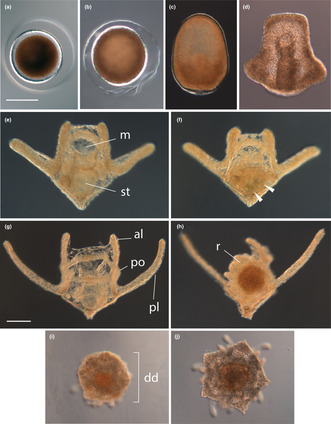

## 1 INTRODUCTION

The evolutionary transition in developmental mode from feeding (planktotrophic) to nonfeeding (lecithotrophic) larvae is widespread across marine invertebrate taxa, and significantly impacts number and size of offspring (Collin & Moran, 2018; Marshall et al., 2012; Strathmann, 1978, 1985). Lineages with feeding larvae have given rise to those with nonfeeding larvae in many marine taxa, including in closely related species (e.g., Byrne, 2006; Collin, 2004; Jeffery et al., 2003; Keever & Hart, 2008; Krug et al., 2015; Pappalardo et al., 2014; Waeschenbach et al., 2012), sometimes with great frequency, for example, at least 15 times in living echinoids (Emlet, 1990). These contrasting patterns signify an evolutionary trade‐off between parental investment per offspring and fecundity: feeding larvae develop from smaller eggs that are produced in far greater numbers than those of related species with nonfeeding development (Strathmann, 1985). The limited provisions of small eggs require planktotrophic larvae to acquire materials through exogenous food to complete metamorphosis. The larger, more lipid‐rich eggs of lecithotrophic larvae contain sufficient material for larvae to create a juvenile without feeding.

An intermediate pattern known as facultative planktotrophy (Chia, 1974; Emlet, 1986; Vance, 1973a), where larvae are not only capable of feeding in the plankton but can also complete metamorphosis without food, is a rare but persistent mode of development that has been observed at least eight times across several marine invertebrate taxa (Table 1; see also Allen & Pernet, 2007). Facultative planktotrophy is often considered an intermediate mode of development in the evolutionary transition between feeding and nonfeeding larvae, but whether or not intermediate modes can maximize reproductive success over evolutionary timescales is still up for debate (Christiansen & Fenchel, 1979; Levitan, 2000; McEdward, 1997; Vance, 1973a, 1973b). Larvae with facultative planktotrophy provide an opportunity to test the effects of larval feeding on several aspects of early life history, including planktonic duration, percent metamorphosis, juvenile size, and energetic reserves in juveniles.

**TABLE 1:** Effects of larval feeding on development of facultative planktotrophs.

Taxa	Development time	Larval survivorship	Juvenile size	References
Echinodermata: Echinoidea				
*Clypeaster rosaceus*	=	=	+	Allen et al. (2006), Emlet (1986)
*Brisaster latifrons*	NM	NM	+	Hart (1996)
Echinodermata: Ophiuroidea				
***Amphiodia* sp. opaque**	**−**	**+**	**+**	**This study**
*Macrophiothrix rhabdota*	−	+	+	Allen and Podolsky (2007)
Mollusca: Gastropoda				
*Adalaria proxima*	−	=	NM	Kempf and Todd (1989)
*Conus pennaceus*	=	=	NM	Perron (1981)
*Phestilla sibogae*	=	+	+	Kempf and Hadfield (1985), Miller (1993)
Annelida: Polychaeta				
*Streblospio benedicti*	NM	NM	NM	Pernet and McArthur (2006)
Arthropoda: Copepoda				
*Tisbe* sp.	−	+	+	Gangur and Marshall (2020)

*Note*: Larval and juvenile metrics are represented as increasing (+), decreasing (−), equal (=), or not measured (NM) because of larval feeding.

Planktonic duration, the time interval between introduction of embryos or larvae to the water column and metamorphosis in the benthos, varies widely across developmental modes of planktonic larvae and affects the composition and distribution of benthic adult populations (Becker et al., 2007; Shanks, 2009; Strathmann, 1985). For feeding larvae, the accumulation of materials necessary for metamorphosis can take weeks to months, whereas nonfeeding larvae can settle in the benthos in hours to days after release (Strathmann, 1987). The larval phase is the dominant dispersal stage for benthic marine invertebrates, and larvae that spend weeks in the plankton tend to disperse further than those that spend only hours in the water column before settlement (Shanks, 2009). Realized dispersal distances are modulated by a complex set of temporal, physical, and behavioral factors, but greater interchange of genetic propagules via feeding larvae tends to lead to greater genetic connectivity between benthic adult populations (reviewed in Cowen & Sponaugle, 2009; Shanks, 2009). The increased dispersal potential of feeding larvae influences biogeography: in cone snails, echinoids, and cowries, species with feeding planktonic larvae have larger geographic ranges when compared to species with nonfeeding development (Emlet, 1995; Kohn & Perron, 1994; Paulay & Meyer, 2006); and in fossil gastropods, there is evidence that planktotrophic development can positively influence geographical distribution and species longevity (Hansen, 1978, 1980; Jablonski, 1986).

Planktonic duration can be mediated by factors both environmental and taxon‐specific, but extended planktonic duration generally increases the likelihood of larval mortality prior to metamorphosis (Rumrill, 1990). Low food availability (Miner et al., 2005; Rendleman et al., 2018; Sewell et al., 2004; Strathmann et al., 1992) and low temperature (O'Connor et al., 2007) can slow larval development in echinoids, as well as facultative planktotrophs from some taxa (Allen & Podolsky, 2007; Paulay et al., 1985). Larvae with longer development times relative to conspecifics experienced lower survivorship in two fishes (Hare & Cowen, 1997; Meekan & Fortier, 1996) and increased mortality from predation in a number of invertebrate species (Cowen & Sponaugle, 2009; Rumrill, 1990). Long planktonic durations also expose larvae to the risk of advection away from suitable habitat for settlement (Pineda et al., 2010), although long‐lived larvae in coastal habitats may not always disperse widely (Shanks, 2009). In these ways, planktonic duration can be tied to the percentage of larvae in a cohort that survive through metamorphosis, hereafter referred to as percent metamorphosis.

Metamorphosis does not promise a new beginning: embryonic and larval experiences can be expressed latently in juvenile and adult quality (Emlet & Sadro, 2006; Pechenik, 2006). Food limitation and prolonged planktonic duration have been shown to negatively influence juvenile size, growth, and survival (reviewed in Pechenik, 2018). In facultative planktotrophs, larval feeding resulted in larger juveniles in echinoderms (Allen & Podolsky, 2007; Emlet, 1986; Hart, 1996) and gastropods (Kempf & Hadfield, 1985; Miller, 1993). Juvenile size is an important life history characteristic because larger juveniles tend to exhibit higher survival, growth, reproduction, and longevity across several species (Marshall et al., 2018).

Here, we present evidence for a facultatively planktotrophic larva of a brittle star, *Amphiodia* sp. opaque (Echinodermata: Ophiuroidea), a previously unknown species that occurs in the northeastern Pacific from Oregon to British Columbia. There is limited documentation of developmental mode within the Ophiuroidea (ca. 12% of ophiuroid species, N. Nakata, unpublished data), but the following developmental modes are known: planktotrophy via an eight‐armed ophiopluteus, facultative planktotrophy, lecithotrophy via a reduced pluteus or vitellaria larva that can be pelagic or demersal, and brooding (Byrne & Selvakumaraswamy, 2002; Hendler, 1991). There are many unresolved relationships within families, but widespread occurrence of developmental diversity suggests frequent transitions from feeding to nonfeeding larvae (Allen & Podolsky, 2007; Lessios & Hendler, 2022; O'Hara et al., 2019). This study utilizes larvae of intermediate mode of development to assess the effect of larval feeding on planktonic duration, percent metamorphosis, juvenile size, and juvenile energetic reserves. Because our experimental individuals were collected as embryos from the plankton and the adults remain unknown (in Oregon), we used DNA barcoding to identify our animals and to reveal their relationship to other ophiuroids in the northeast Pacific.

## 2 MATERIALS AND METHODS

### 2.1 Molecular identification of embryos and larvae

To investigate species identity of the embryonic morphotypes, we froze one embryo from each year's cohort of larvae collected for experimentation (2019–2021, see section 2.2) at −20°C in a small volume of seawater. We compared the resulting sequences with those of approximately a dozen larvae of the same morphotype collected over the last decade, as well as with sequences for adults and larvae of other amphiurids from the northeast Pacific. Genomic DNA was extracted with the chelex‐based InstaGene™ Matrix (Bio‐Rad) and fragments of *cytochrome c oxidase subunit I* (*COI*) were amplified and sequenced using the primers jgLCO1490 and jgHCO2198 (Geller et al., 2013). PCR amplification reactions were performed in a 20 μL total reaction volume that included 11.4 μL nuclease‐free water, 4 μL 5X Green Buffer, 0.4 μL dNTP 10 mM, 0.2 μL GoTaq Polymerase (Promega), and 1 μL each of forward and reverse primers. PCR conditions were as follows: initial step 95°C for 2 min, followed by 34 cycles of denaturation at 95°C for 40 s, annealing at 45 or 48°C for 40 s, and extension at 72°C for 1 min, followed by a final extension at 72°C for 2 min. PCR products were cleaned up using the Wizard SV Gel and PCR Clean up System (Promega) prior to Sanger sequencing (Sequetech). Barcode sequences were compared to GenBank (www.ncbi.nlm.nih.gov/genbank/), BOLD (Ratnasingham & Hebert, 2007), and our unpublished dataset of ophiuroid sequences using the BLAST function in Geneious Prime (https://www.geneious.com). We aligned *COI* sequences of *Amphiurid* spp. of the northeast Pacific (Table 2) using the Geneious MAFFT plug‐in (Katoh & Standley, 2013) created a maximum likelihood tree using the PhyML plug‐in with 100 bootstraps and based on the HKY85 model (Guindon et al., 2010; Hasegawa et al., 1985).

**TABLE 2:** Collection and accession data for specimens included in tree (Figure 2).

Species ID	Specimen code	Life stage	Collection location, State/Province	Accession number
*Amphichondrius granulatus*	Agran1	Adult	Catalina Is., CA	OOPH005-18
	Agran2	Adult	Catalina Is., CA	OOPH006-18
*Amphipholis pugetana*	Ampu1	Adult	Cape Arago, OR	OOPH003-18
	Ampu2	Adult	Cape Arago, OR	OOPH004-18
	Or43‐12913	Larva	Charleston, OR	OLAB015-22
	Or811‐12115	Larva	Charleston, OR	OLAB016-22
*Amphiodia occidentalis*	Amoc2	Adult	Charleston, OR	OOPH002-18
	AoMc1a	Adult	Charleston, OR	OOPH020-22
	Amoc1	Adult	Charleston, OR	OOPH001-18
	AoMc2a	Adult	Charleston, OR	OOPH022-22
*Amphiodia* sp. sensu Emlet (2006)	FHLAmphioegg1	Egg	Orcas Island, WA	OLAB045-22
	OrR7	Larva	Charleston, OR	OLAB031-22
	Or8P‐121812	Larva	Charleston, OR	OLAB033-22
	MMB17	Adult	Charleston, OR	OOPH032-22
***Amphiodia* sp. opaque**	BFHL 4167	Adult	Boundary Bay, WA	BBPS564-19
	Orop3a	Larva	Charleston, OR	OLAB001-22
	Opq4	Larva	Charleston, OR	OLAB006-22
	Oop1	Larva	Charleston, OR	OLAB007-22
	QHAK‐00565	Larva	Quadra Island, BC	QHAK711-21
	Opq2	Larva	Charleston, OR	OLAB004-22
	opaque 11‐7‐18	Larva	Charleston, OR	OOPH029-22
	Opq1 FHL	Larva	Friday Harbor, WA	OLAB047-22
*Amphiodia urtica*	Or988	Juvenile	Charleston, OR	OLAB028-22
	A.urt adt 4‐23‐19	Adult	Cape Arago, OR	OOPH026-22
	DISA835‐19	Adult	Los Angeles, CA	DISA835-19
	HM542069	Adult	Bamfield, BC	*HM542069
	urtica 11‐5‐18	Larva	Charleston, OR	OOPH027-22
	CCDB‐31778 B01	Adult	Dana Point, CA	ECHCA108-18
	KU495782	Adult	Queen Charlotte Is., BC	*KU495782

*Note*: Accession numbers refer to records in BOLD (Ratnasingham & Hebert, 2007), unless marked by an asterisk (*), indicating that a record is from GenBank (Benson et al., 2017).

### 2.2 Larval cultures and data collection

**FIGURE 1:**
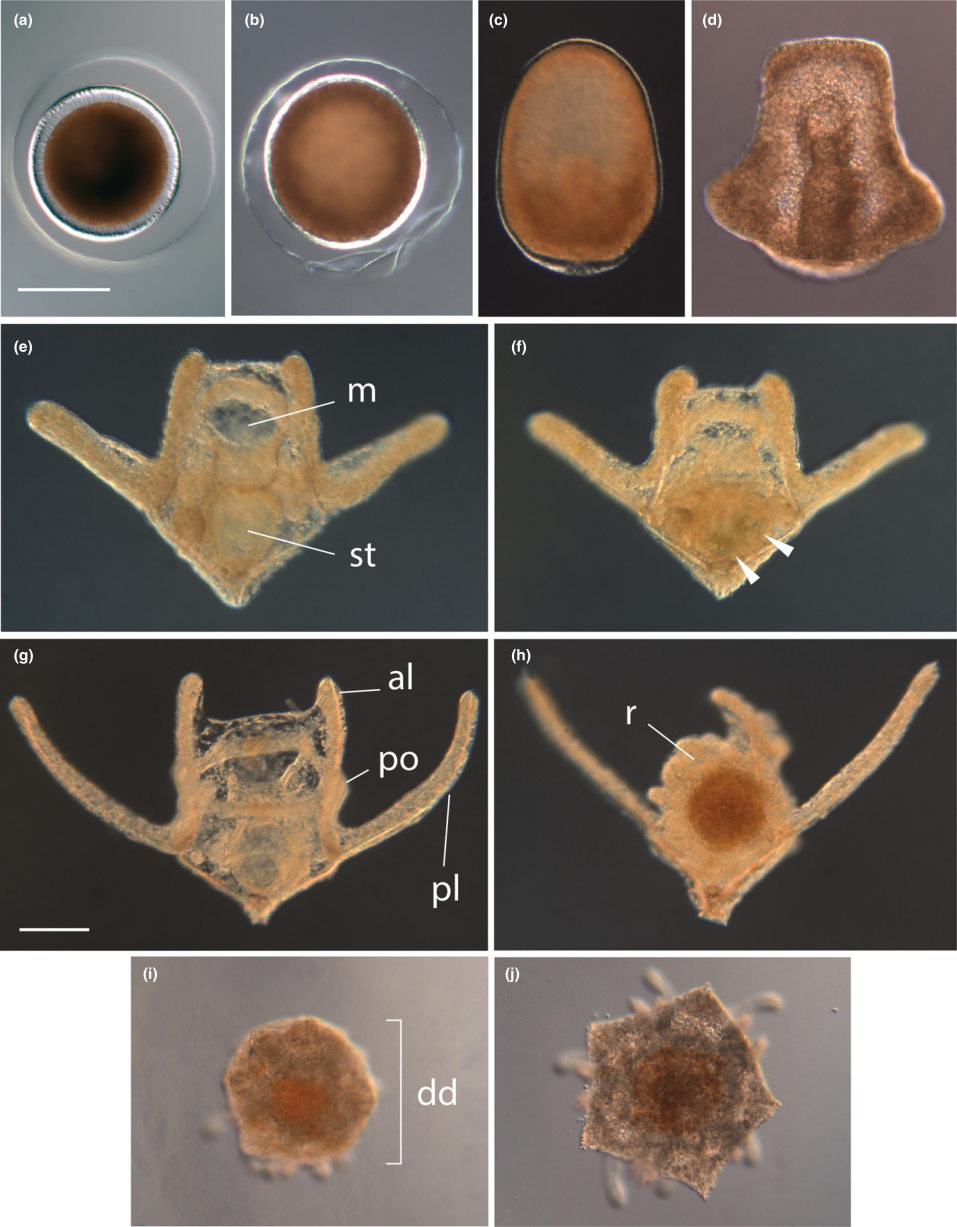
*Amphiodia* sp. opaque (a) fertilized egg, (b) unhatched blastula, (c) gastrula, (d) early pluteus 3 days post spawn (dps), (e) reduced pluteus 6 dps from no‐food treatment, with mouth (m) and empty stomach (st), (f) reduced pluteus 6 dps from food treatment, with algal food visible in the stomach (arrowheads), (g) pluteus at 10 dps from no‐food treatment, with three pairs of arms: anterolateral (al), postoral (po), and posterolateral (pl), (h) pluteus 10 dps from food treatment, with juvenile rudiment (r), (i) juvenile from no‐food treatment with disk diameter (dd), and (j) juvenile from food treatment. Scale bars are 100 μm; same scale for (a–f) and for (g–j).

Adults of *Amphiodia* sp. opaque have not yet been found in Oregon. We obtained late blastulae, hatched gastrulae, and larvae of *Amphiodia* sp. from plankton tows collected with 130‐μm mesh net in the Coos Bay estuary (Oregon) approximately 3 km from the entrance to the Pacific Ocean (Charleston Marina: 43°21.2′ N, 124°20′ W). We collected plankton daily, approximately 1 h before high tide and examined our catch within 2 h using a stereomicroscope. On several occasions, the bright orange embryos characteristic of *Amphiodia* sp. opaque (Figure 1a,b) were abundant enough to obtain sufficient material for experimentation. In 2020, we collected approximately 120 blastulae or gastrulae, ~1 day post spawn (dps), on February 23 and 100 more of similar stage on February 24. Because of their similar stages of development, embryos from each day represented separate spawning events 1 day apart. In 2021, we collected 240 embryos of similar stage on March 7. In 2019, no embryos were found, but we collected approximately 80 ophioplutei on January 28 and 29. These larvae had either formed juvenile rudiments or did so within a week of capture and were analyzed for juvenile size and starvation time as a “wild” treatment. The ophiuroid hydrocoele rudiment wraps around the larval esophagus and marks the cessation of larval feeding; therefore, we considered larvae with rudiments to have completed or nearly completed larval feeding in their natural environment prior to their collection and monitoring in the laboratory. Wild ophioplutei and rudiment‐stage larvae were kept in filtered sea water (FSW) for the remainder of their development. We did not manipulate microalgal food or estimate planktonic duration for larvae collected in 2019 because we could not determine their prior history in the plankton.

### 2.3 Feeding experiments

For experimental manipulations of microalgal food, we kept embryos (collected in 2020 or 2021) in FSW until just before the formation of the mouth, approximately 2 days after collection. At this point, we haphazardly divided early plutei into replicate finger bowls each with 10 larvae per 30 mL (2020) or 17 larvae per 51 mL (2021) for a standard density of 0.3 larva per mL. We randomly assigned bowls to “food” or “no‐food” treatments and kept them in an incubator at 15°C. We fed larvae in the food treatment a tripartite microalgal diet composed of two parts by volume of *Rhodomonas lens*, to one part each of *Dunaliella tertiolecta* and *Isochrysis galbana* at a combined concentration of 5000 cells per mL. Larvae in the no‐food treatment were kept in FSW alone. Small flakes of cetyl alcohol were added to all cultures to prevent larvae from perishing in the air–water interface.

We collected data on stages of larval development every 2 or 3 days when FSW and microalgal food were refreshed. We categorized larvae visually as (1) ophiopluteus, (2) rudiment‐stage larva, or (3) juvenile (Figure 1). Developmental stages were defined as follows: ophioplutei had up to six larval arms and an open mouth; rudiment‐stage larvae had a pair of posterolateral arms, the right anterolateral arm and a well‐developed rudiment that occluded the larval mouth; and juveniles lacked larval arms including the larval skeleton and ciliary band, and all locomotion was by their podia. Planktonic duration was defined as the number of days from hatching—estimated as 1 day prior to the blastula stage in which they were collected—until they were scored as juveniles. We defined percent metamorphosis as the total number of juveniles produced relative to the initial number of larvae from a given bowl. When we found new juveniles, we removed them from the larval culture bowl and put each individual in its own 35 mm Petri dish with FSW. We kept juveniles in FSW without food at 15°C, observing them every 2–3 days when FSW was changed. A juvenile was considered dead when podia did not move, even following water agitation and the juvenile did not hold onto the dish bottom.

### 2.4 Analysis

We conducted all statistical analyses in the R environment v3.6.0 (R Core Team, 2022), and visualized plots with the package “ggplot2” (Wickham, 2016).

For each statistical test to follow, aside from the survival analysis of juveniles, we considered the finger bowl to be the experimental unit. We calculated the percent of larvae that successfully metamorphosed into juveniles (hereafter percent metamorphosis), and mean values for planktonic duration, juvenile size, and juvenile starvation time for each bowl. We calculated survival statistics in two ways: by bowl (Figure 6b) or pooled within treatments and years in survival analysis (Figure 7).

To quantify the effect of larval food on percent metamorphosis, we compared between treatments, keeping years separate. As values for percent metamorphosis were not normally distributed and did not have equal variances (Shapiro–Wilk, *p* = .015; Levene's test, *p* = .004), we used a nonparametric Kruskal–Wallis test, followed by a Dunn's test for pairwise comparisons.

We used aboral surface area of the disk as our value for juvenile size. We measured disk diameter (dd, Figure 1i), the length from one arm tip through the center of the disk to the opposite interradius, using the ocular micrometer on the dissecting microscope (dh, Figure 1i), measured to the nearest 20 μm. Using the disk diameter as the height of a regular pentagon (*h*, Equation 1), we solved for the length of a single side (*a*) in order to calculate the area of the pentagonal juvenile disc (*A*, Equation 2).

(1) $$h=a\times \sqrt{5+2\sqrt{5}}/2$$

(2) $$A=a^{2}\times \sqrt{25+10\sqrt{5}}/4$$

The data for juvenile aboral surface area did not satisfy the assumption of normality (Shapiro–Wilk, 2020 *p* = .001, 2021 *p* = .006), but variances were not significantly different (Levene's test, 2020 *p* = .97, 2021 *p* = .09). To determine if the presence of microalgal food had an effect on juvenile size, we compared aboral surface area across treatments using a nonparametric Kruskal–Wallis test, followed by a post hoc Dunn's test.

To determine whether larvae that developed more quickly also produced larger juveniles, we modeled the association between planktonic duration and juvenile aboral surface area using a set of generalized linear models (GLMs) with a Gaussian family, including combinations of potential covariates including treatment and year.

To examine the relationship between juvenile size and survival, we modeled the association between juvenile aboral surface area and time to starvation using a set of GLMs and combinations of covariates treatment, year, and their interaction. As we predict time to juvenile starvation represents a waiting time, we used GLMs with a gamma distribution. We assessed model fit using Akaike's and Bayesian Information Criteria (Table S4), with the lowest value indicating the best fit.

To assess differences in juvenile survival time between treatments and years, we used survival analysis. The survival time data violated the assumptions of parametric statistics, rendering traditional tests inappropriate for their analysis. Such data can be analyzed using nonparametric survival analysis (Kleinbaum & Klein, 2012; Moore, 2016), a collection of statistical procedures for which the outcome variable of interest is time until an event occurs. Juvenile starvation times were analyzed using survival analysis in the R package “survival” (Therneau, 2020) and visualized with the package “survminer” (Kassambara et al., 2020).

Most survival analyses must contend with censoring, which occurs when we have some information about individual survival time, but we do not know the survival time exactly. Our data on time to juvenile starvation are “interval‐censored,” because counts were done at intervals when water changes happened every 2 or 3 days. The exact time of metamorphosis or death is unknown and can only be placed within a specified window of time. Censoring can also occur when an individual withdraws from the study prior to the time of the event. In our analysis of juvenile starvation, the event of interest was death, and no juveniles were censored as all were followed until the time interval of the event. Survival curves for juvenile death probabilities were compared using a nonparametric log‐rank test.

## 3 RESULTS

### 3.1 Species identity and occurrence of larvae

**FIGURE 2:**
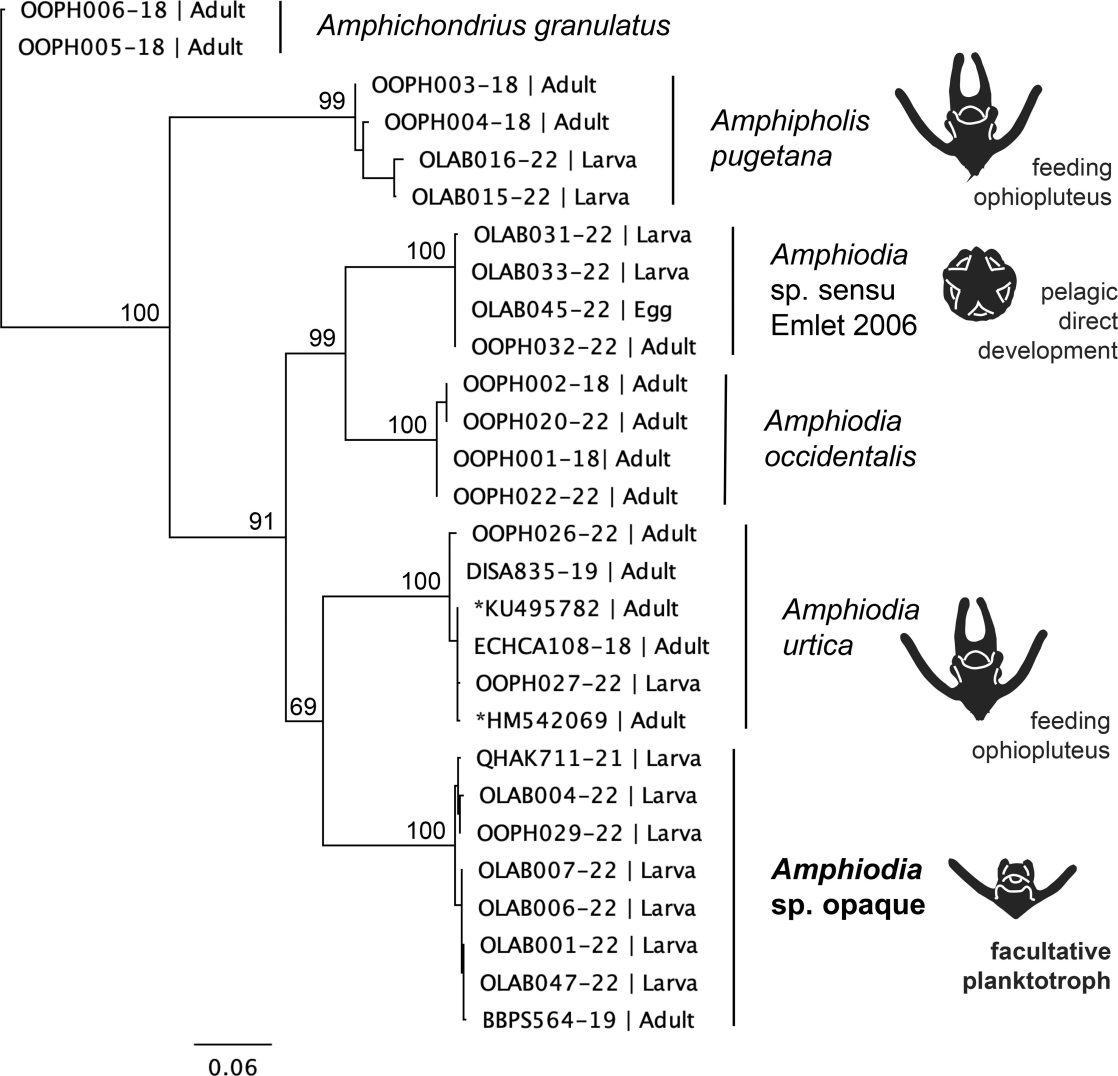
Maximum likelihood tree of *COI* sequences from adult and larval *Amphiurid* spp. in the northeastern Pacific, constructed using the PhyML plug‐in in Geneious. Sequences from GenBank (*) and BOLD are labeled with accession number (and see Table 2). Development modes are indicated on the right, with characteristic larvae depicted. Bootstrap values are shown next to nodes.

The opaque orange larvae studied here belong to an undescribed species of *Amphiodia* that occurs at least from southern Oregon to British Columbia. On many occasions since March 2003, we have collected eggs, unhatched and hatched embryos, and larvae from Oregon plankton that we assigned, by color and morphology to *Amphiodia* sp. opaque. These samples were collected between January and April, but on one occasion, the embryos were collected in November (2018). We also collected these larvae in the Salish Sea (Friday Harbor, Washington) in August of 2019 and September of 2020. We have generated *COI*‐barcodes for more than 15 specimens collected from 2013 to 2019 that we assigned to *Amphiodia* sp. opaque, and their sequences are 98+ % similar (Figure 2). During the times we collected these embryos and larvae, we never found similar orange embryos or larvae that had different barcodes and were other species.

The sequences of our larvae are a close match (>98% pairwise nucleotide similarity) to that of a juvenile amphiurid specimen (2.5 mm disk diameter) collected from Boundary Bay WA (BOLD specimen record: BBPS564‐19). The juvenile is clearly a member of the genus *Amphiodia*, because it has the appropriate pattern of three similarly sized oral papillae per jaw. In BOLD, we found another close match (>99%) to a larva with the same morphology from Hyacinthe Bay, Quadra Island, British Columbia (BOLD specimen record: QHAK711‐21, Figure 2).

A neighbor‐joining tree of *COI* sequences from larvae and adults of other amphiurid species known from the NE Pacific shows *Amphiodia* sp. opaque as the sister species to *A. urtica* which has obligately feeding larvae (Schiff & Bergen, 1996, N. Nakata, unpublished data; Figure 2). Together these clades are sister to a species complex of *A. occidentalis* (R. Emlet, unpublished data).

### 3.2 Larval developmental mode

Larvae of *Amphiodia* sp. opaque develop from eggs of moderate size (140 μm) and are facultative planktotrophs (Figure 1): larvae can feed but do not require food for metamorphosis into juveniles. While percent metamorphosis did differ between years, some larvae developed into juveniles in the absence of food. We observed larvae from unfed culture with empty stomachs, and those from fed cultures with stomachs full of microalgal food (Figure 1e,f). Furthermore, aspects of larval and juvenile performance differed according to larval food treatment (Table 3).

**TABLE 3:** Summary of traits for larval and juvenile *Amphiodia* sp. opaque.

Year	Treatment	Total larvae/no. replicate bowls	Planktonic duration (days)	Juv., *n*	Percent metamorphosis	Juv. aboral surface area (mm^2^)	Time to starvation (days)
2019	Wild	80/7	–	46	–	0.032 ± 0.001	0–6–38
2020	Food	110/11	15–17–21	88	80 ± 3	0.046 ± 0.001	2–59–104
	No‐food	110/11	20–23.5–30	16	14 ± 6	0.030 ± 0.001	0–48.5–76
2021	Food	120/7	13–13–19	54	45 ± 4	0.047 ± 0.001	2–23–65
	No‐food	120/7	15–22–33	64	53 ± 3	0.032 ± 0.001	2–18.5–54

*Note*: Larvae were raised with and without microalgal food in 2020 and 2021 and collected as late‐stage plutei in 2019. Planktonic duration is given for all larvae that completed metamorphosis. Planktonic duration and time to juvenile starvation are time data and are listed as minimum, median, and maximum. Percent metamorphosis and juvenile aboral surface area are given as means ± standard error for replicate bowls and for individuals, respectively.

### 3.3 Effects of larval food on developmental timing

**FIGURE 3:**
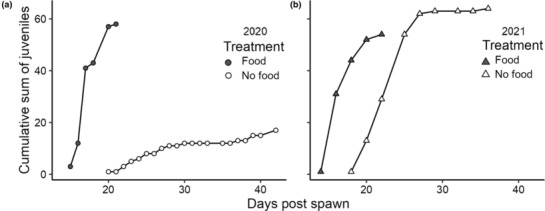
Cumulative sum of juveniles over time by treatment and year: (a) 2020 and (b) 2021. Each year had the same initial number of larvae in each treatment (2020 *n* = 110 per treatment, 2021 *n* = 120). Final time points represent date of discovery of the last juvenile in that treatment.

Fed larvae reached metamorphosis more quickly than unfed larvae (Figure 3). Planktonic duration differed between years but was about a week shorter for fed larvae than for larvae raised in FSW (2020 median 17 vs. 23.5 days; 2021 median 13 vs. 22 days; see Table 3).

### 3.4 Effects of larval food on percent metamorphosis

**FIGURE 4:**
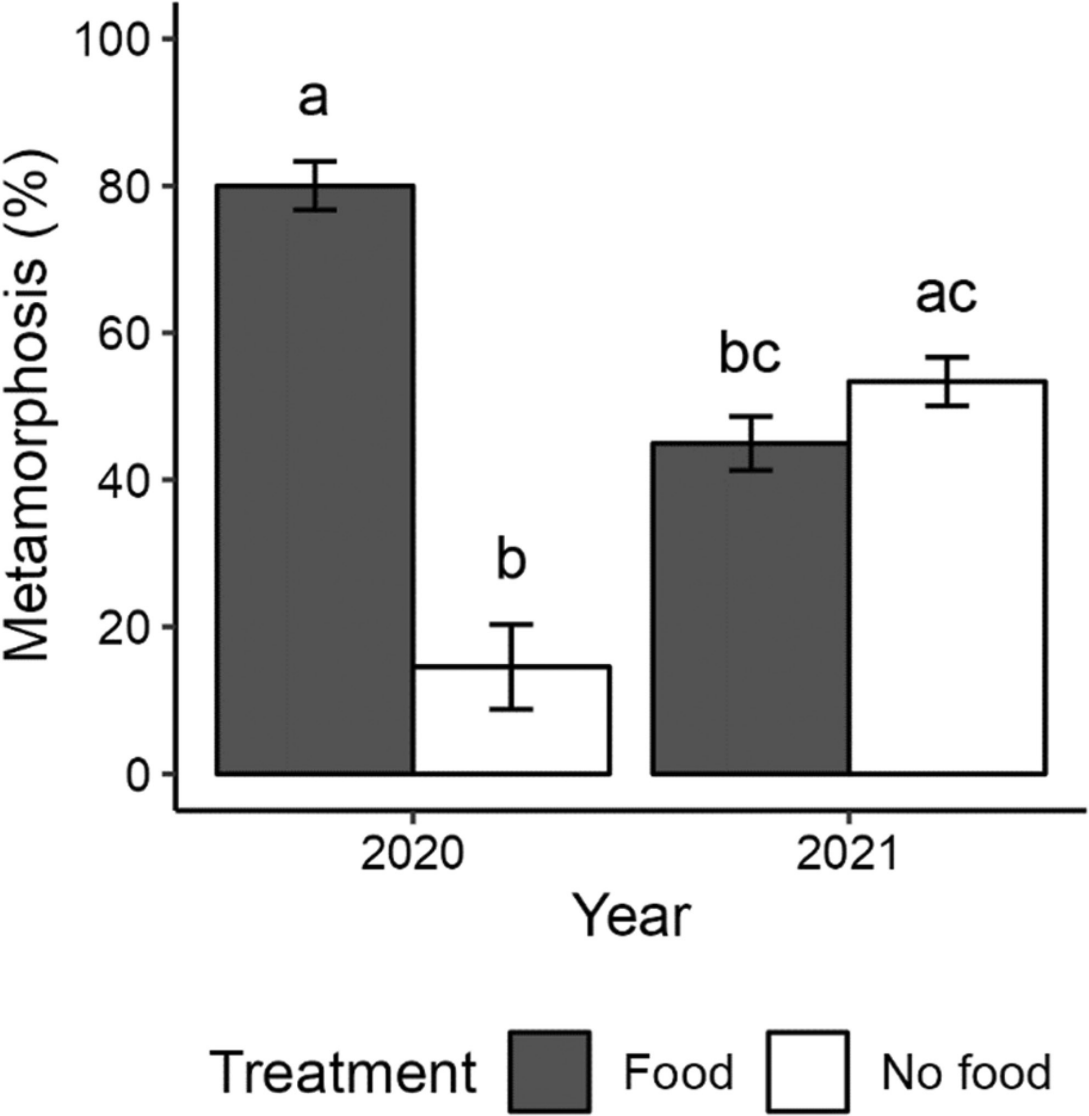
Bar plots of percent metamorphosis (= no. juveniles/initial no. larvae) averaged across experimental bowls for years 2020 (initial larvae *n* = 220/22 bowls) and 2021 (*n* = 240/14 bowls). Error bars represent standard error (SE). Lowercase letters above bars represent significant differences in pairwise comparisons.

The effect of treatment on the percent of larvae that completed metamorphosis differed significantly in 2020 but not in 2021 (across all treatments and years: Kruskal–Wallis test: *χ* ^2^ = 29.25, df = 3, *p* < .001; Figure 4). Pairwise comparisons show a significant difference between treatments in 2020 (Dunn's test: adj. *p* < .001, Table S1), when food had a strong effect: 80% of fed larvae created a juvenile whereas only 15% of unfed larvae were able to do so (Figure 4). By contrast, in 2021, slightly more juveniles resulted from cultures without larval food (food: 45%, no‐food: 53%), and percent metamorphosis was not statistically different between treatments (*p* = .44).

### 3.5 Effects of larval food on juvenile size

**FIGURE 5:**
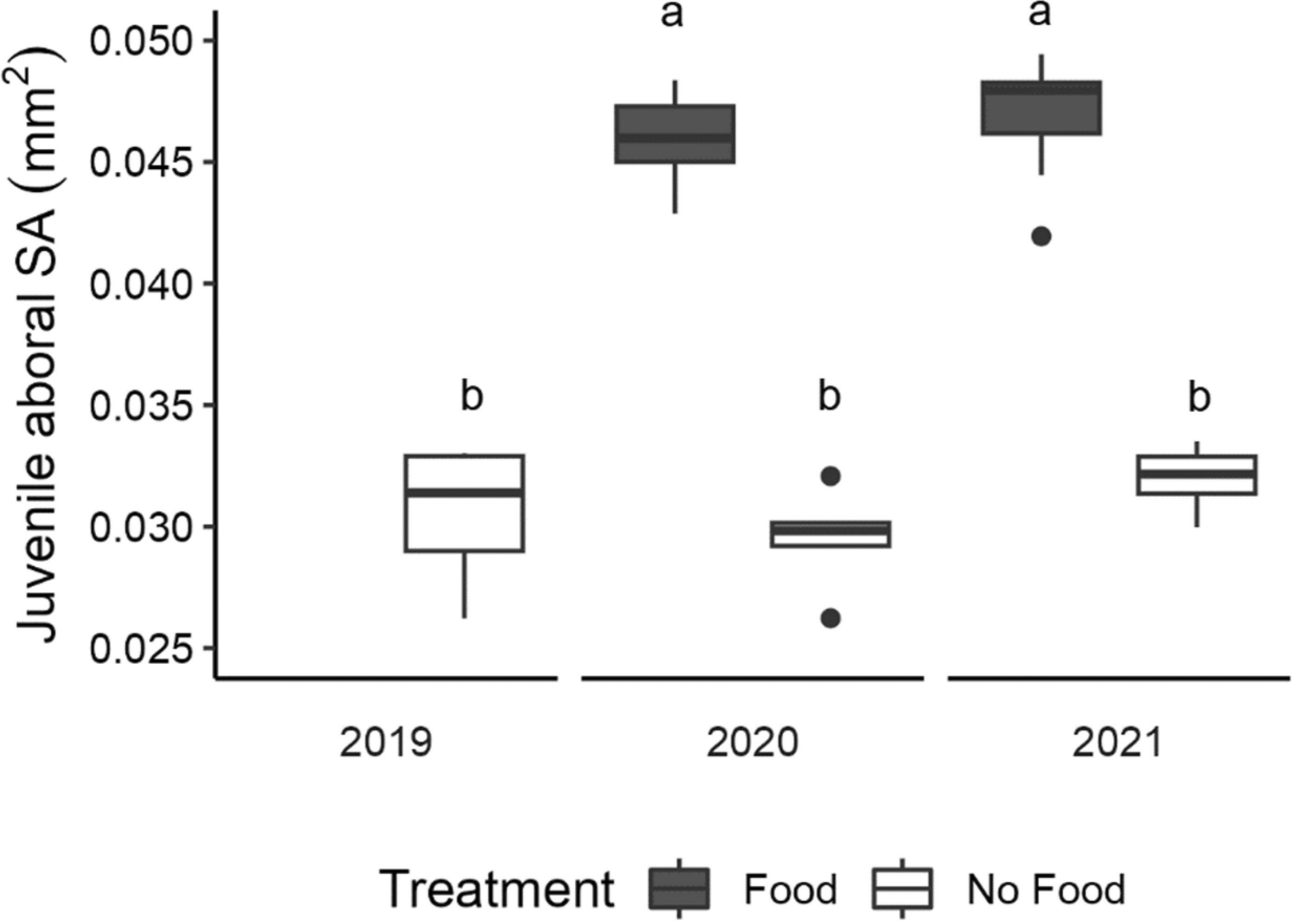
Boxplot of juvenile aboral surface area (SA) at metamorphosis by food treatment, pooled across years. Lowercase letters above boxplots represent significant differences in pairwise comparisons.

Juvenile sizes measured as aboral surface area were significantly different between treatments in both years (Kruskal–Wallis test: *χ* ^2^ = 26.149, df = 4, *p* < .0001). Pairwise comparisons showed that juveniles from fed larvae were larger than juveniles from unfed larvae in both years. Juveniles from wild larvae were not significantly different in size from juveniles from the no‐food treatment (Dunn's test, Table S2; Figure 5).

**FIGURE 6:**
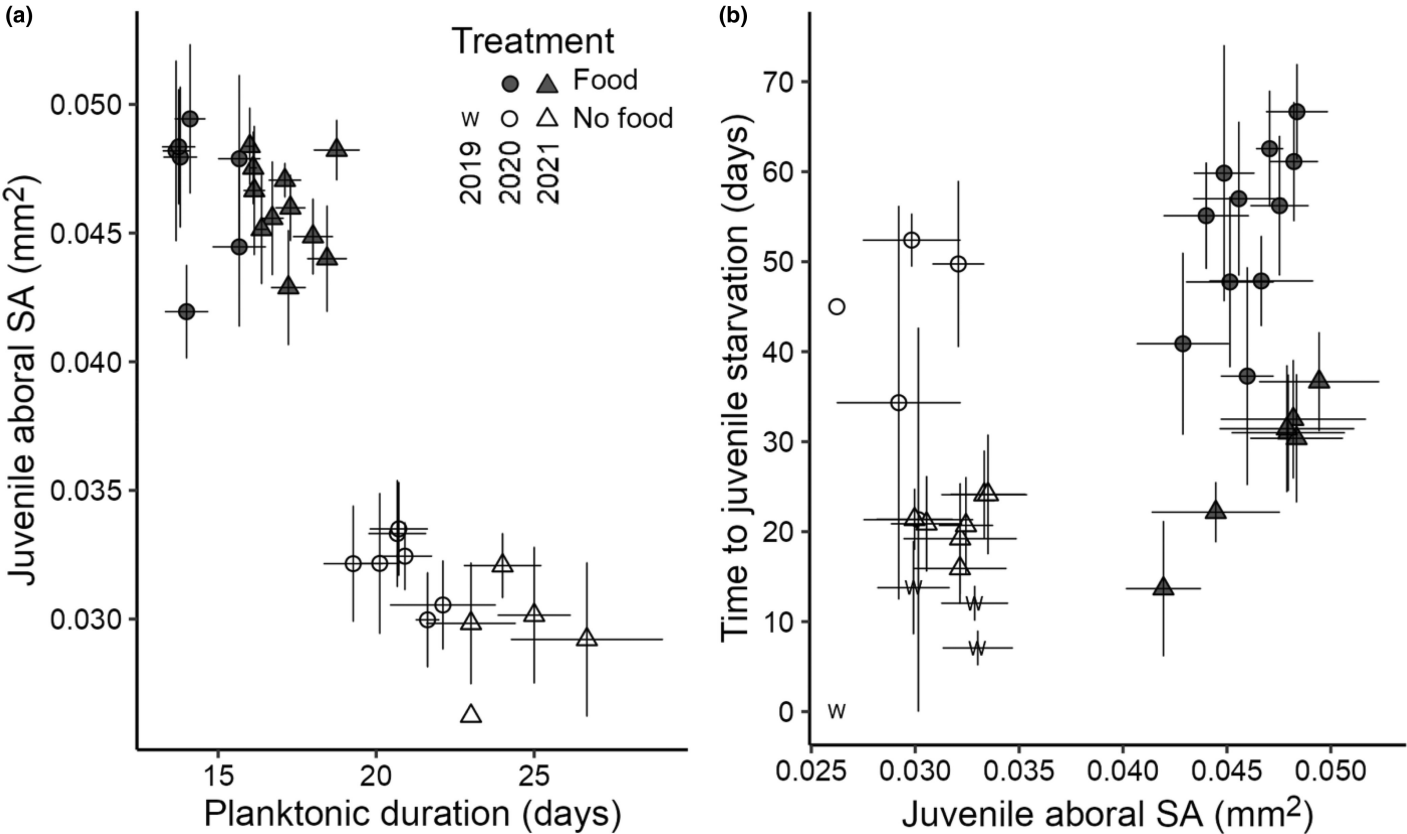
Scatterplots of (a) planktonic duration by juvenile size, and (b) juvenile aboral surface area (SA) by time to juvenile starvation. Points are mean values for replicate finger bowls (larval culture containers) and bars are standard error. Treatment is coded by color (food: dark gray, no‐food: white, wild: “w”) and experimental year by shape (2020: circle, 2021: triangle, wild: “w”).

We modeled the association between planktonic duration and juvenile aboral surface area (Figure 6a) using a series of GLMs and found that the best fit model included only treatment as a covariate (AIC = −288.3, Table S3).

### 3.6 Effects of larval food on juvenile survival

The survival time in juveniles that received food as larvae was longer compared to those that did not receive food, but survival times varied between years (2020 median 59 days [*n* = 88 vs. 48.5 days, *n* = 16; 2021 median 23 days, *n* = 54 vs. 18.5 days, *n* = 64, Table 3]). Juveniles that resulted from wild‐caught larvae had relatively short survival times under starvation conditions (median 6 days, *n* = 80).

Bigger juveniles tended to survive longer, and juveniles from fed larvae had greater surface areas than those from larvae that did not receive food (Figure 6b). Juvenile aboral surface area was positively associated with days to juvenile starvation and the best‐fit model included an interaction between treatment and year (GLM, AIC 1672.1; Table S4). Treatment (food, no‐food) and experimental year (2020, 2021) were both significant covariates (*p* = .02 and *p* < .001, respectively).

**FIGURE 7:**
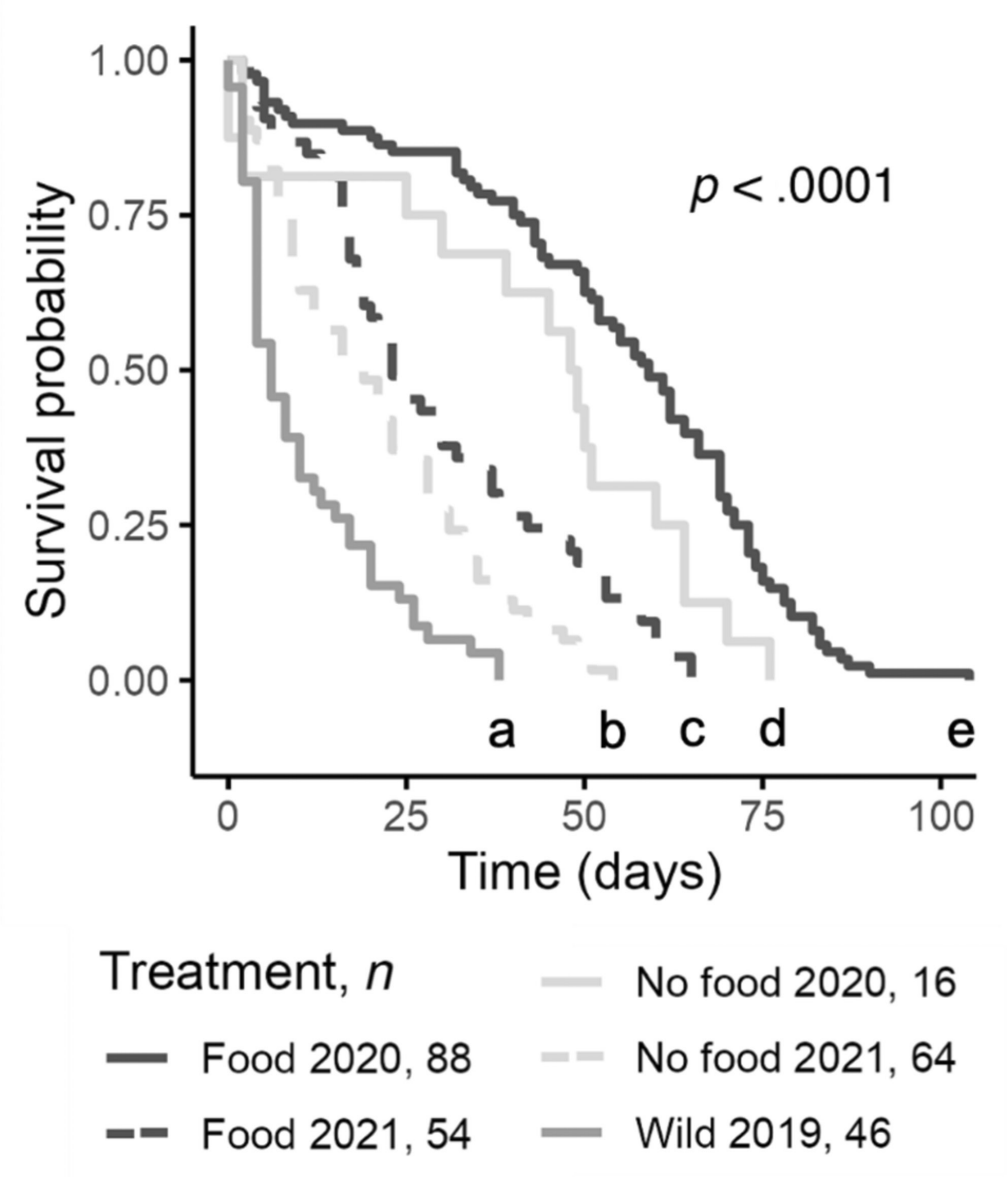
Kaplan–Meier survival curves for *Amphiodia* juveniles according to larval food treatment and year. No juveniles were censored as they were followed until the time of the event, death. Lowercase letters below survival curves indicate significantly different pairwise comparisons.

The survival curves for each treatment and year were significantly different from each other (log‐rank test, *p* < .0001) and were significantly different in each pairwise comparison (*p* < .0001; Figure 7).

## 4 DISCUSSION

### 4.1 Species identity

Through DNA barcoding of the *COI* gene, we found a single species‐level match (>98% pairwise nucleotide similarity) to our *Amphiodia* larvae in a juvenile specimen from Boundary Bay, WA (BBPS564‐19, provided by G. Paulay), which was difficult to assign to species from its morphology. The specimen was small (2.5 mm disk diameter) and the adult characteristics necessary for identification may have been absent or reduced, as they often are in small specimens (Stöhr, 2005). The specimen had three oral papillae of equal size and spacing, as is characteristic of *Amphiodia*. Its oral shields are pentagonal in shape, and the arm spines are tapered to a point like in *A. urtica*, but the radial shields are approximately two times as long as they are wide, the dorsal arm plates are oblong and the ventral arms plates are squarish like in *A. occidentalis* (Lambert & Austin, 2007). Furthermore, molecular data for *COI* delineates this specimen from adults of *A. occidentalis* or *A. urtica* (Figure 2). Rather than signifying a new species, the lack of a molecular match with morphological identification may be due to the limited genetic sampling of the genus *Amphiodia* (8 of 34 species, N. Nakata, unpublished data) and of amphiurids of the northeast Pacific (5 of 12 species, N. Nakata, unpublished data).

### 4.2 Larval morphology and developmental mode

Using feeding assays, we confirmed that *Amphiodia* sp. opaque is a facultative planktotroph. *Amphiodia* sp. opaque has eggs of moderate size (140 μm), which is consistent with other ophiuroids with abbreviated development (Hendler, 1991). Larvae of this species developed into juveniles in the absence of microalgal food but were still capable of planktonic feeding. Percent metamorphosis varied across treatments and study years, but 15%–53% of larvae given no microalgal food successfully completed metamorphosis, supporting our diagnosis of facultative planktotrophy.

This developmental mode cannot be diagnosed from larval morphology alone, because larvae retain the structures necessary for feeding. Larvae must be cultured in the presence and absence of microalgal food to determine developmental mode. We did suspect facultative planktotrophy of this larva because it was opaque orange in color and has a reduced pluteus morphology (Hendler, 1975), as determined by comparison with a sympatric congener *A. urtica*, which has a transparent, planktotrophic larva with eight arms. The posterolateral arms are reduced in length relative to those of *A. urtica*, and the posterodorsal arms are highly reduced or absent, resulting in six larval arms instead of eight. Larvae of *Amphiodia* sp. opaque have other features that are associated with evolutionary transitions in developmental pattern, including an egg of intermediate size (ca. 140 μm) and intermediate planktonic duration.

Facultative planktotrophy has been observed in six other marine invertebrates (Table 1). *Amphiodia* sp. opaque is the second facultative planktotroph to be described from the Ophiuroidea and the first from the family Amphiuridae. Only one ophiuroid was previously known to have this developmental pattern: *Macrophiothrix rhabdota* (Ophiotrichidae) develops via an 8‐arm pluteus. The family Ophiotrichidae diverged from Amphiuridae approximately 200 Mya (O'Hara et al., 2017), meaning *Amphiodia* sp. opaque represents an independent evolution of the facultative planktotroph phenotype. Food‐limited growth is common for larvae of benthic invertebrates (Paulay et al., 1985), and facultative planktotrophy may have evolved as a bet‐hedging strategy to increase the number of resulting juveniles in regions or spawning times when planktonic food availability is low.

### 4.3 Development time

Fed larvae developed more quickly than larvae that did not receive food. Larvae in the food treatment completed metamorphosis in fewer days than their starved peers (Figure 2). This is consistent with echinoids with feeding larvae, which are known to take longer to form their rudiment in no‐ and low‐food conditions (Miner et al., 2005; Sewell et al., 2004; Strathmann et al., 1992).

Compared to congenerics of contrasting development modes, *Amphiodia* sp. opaque has an intermediate planktonic duration (medians of 13–17 days when fed). The planktotroph *Amphiodia urtica* has a median planktonic duration of 20 days (*n* = 10 larvae collected at different times, N. Nakata, unpublished data) and a congeneric, pelagic, direct‐developer may be planktonic for 8 days at 15°C (Emlet, 2006).

The availability of food for larvae does not always decrease time to metamorphosis in facultative planktotrophs, For example, there was no difference between fed and unfed treatments in the echinoid *Clypeaster rosaceus* or the gastropods *Conus pennaceus* and *Phestilla sibogae* (Emlet, 1986; Kempf & Hadfield, 1985; Perron, 1981). In the ophiuroid *Macrophiothtrix rhabdota*, planktonic duration was decreased in the presence of food (Allen & Podolsky, 2007). Shorter planktonic interval may limit mortality from predation in the plankton (Rumrill, 1990) and may reduce capacity for dispersal in species with shorter developmental times (Hendler, 1991; Shanks, 2009).

We interpret larval developmental times as dependent on the accumulation of energetic reserves necessary for metamorphosis and juvenile life, but other factors may have contributed to planktonic durations observed in this study. Little is known about cues for competency or settlement in ophiuroids (Hendler, 1991; Hodin et al., 2015). Furthermore, ophiuroids are known to be capable of undergoing metamorphosis in the plankton and continuing to ride ocean currents as juveniles (Hendler et al., 1999), indicating that some species may not require benthic cues to begin metamorphosis once sufficient food has been consumed in the plankton.

### 4.4 Percent metamorphosis

The effect of larval feeding on the proportion of larvae that completed metamorphosis was different across years for *Amphiodia* sp. opaque. In 2020, food had a strong effect on the proportion of larvae able to complete metamorphosis, as was true for the other facultatively planktotrophic ophiuroid (Allen & Podolsky, 2007). In 2021, percent metamorphosis was not significantly different between treatments and slightly more juveniles resulted from the no‐food treatment (Figures 2 and 3). Among facultative planktotrophs, larval feeding did not affect percent metamorphosis (referred to as larval survival) in an echinoid (Emlet, 1986) and two gastropods (Kempf & Todd, 1989; Perron, 1981).

It is possible that the observed variation between cohorts resulted from differences in culture conditions between years, or from intrapopulation or interannual variation in parental investment and developmental regimes. As all embryos originated from the plankton, we are unable to determine if differences between cohorts reflect variation in maternal provisioning due to nutrition or genetic variation between populations.

### 4.5 Juvenile size

We observed that fed larvae of *Amphiodia* sp. opaque produced juveniles that were ca. 50% larger in surface area than those of starved larvae (Table 3; Figure 5). Increased juvenile size as a consequence of larval feeding has been observed in other facultative planktotrophs (Allen & Podolsky, 2007; Emlet, 1986; Hart, 1996; Kempf & Hadfield, 1985; Miller, 1993). Juveniles of *Amphiodia* sp. opaque from the fed treatment were slightly smaller than those of a sympatric congener with feeding larvae, *A. urtica* (juvenile aboral surface area 0.047 mm^2^, *n* = 18; N. Nakata, unpublished data).

Advanced larvae collected from wild plankton (2019) produced juveniles that were not significantly different in size from those from the experimental no‐food treatment. This suggests that these larvae had limited opportunity to feed in their natural environment (and they were not fed in the laboratory). Even low‐food conditions can lead to smaller juveniles in a calyptraeid gastropod (Chiu et al., 2007, 2008). Evidence from a barnacle has shown that even food deprivation during a portion of the larval life can lead to smaller juveniles with the early stages being the most important (Emlet & Sadro, 2006).

### 4.6 Juvenile survival

Juveniles of fed larvae lived longer than those from larvae raised without food, suggesting that they gained greater energetic reserves due to larval feeding. However, juvenile survival times differed among treatments and experimental years (Figure 7). Juveniles from both treatments in 2021 had shorter survival times than juveniles from either treatment in 2020. Interestingly, the juveniles that resulted from wild‐caught larvae from 2019 fared the poorest of all. They were no different in size from juveniles of laboratory‐reared larvae that received no food (Figure 5), but they died from starvation more quickly (Figures 6b and 7). This may have been the result of variation in egg composition or size, but whether that was due to intraspecific genetic variation, local environmental deficiencies that lowered maternal condition, or some combination was not determined.

## 5 CONCLUSION

In this study, we tested the influence of larval feeding in an unidentified facultatively planktotrophic larva, *Amphiodia* sp. opaque. We know this animal only from its larva. We utilized phylogenetic analysis in an attempt to determine species identification and to compare it with closely related ophiuroids from the NE Pacific. Nevertheless, we were able to conduct a series of experiments that showed clear benefits of larval feeding across multiple life history characters: larvae that received food developed more quickly, experienced higher rates of metamorphosis, and produced larger juveniles that evaded starvation conditions for longer than those of larvae that received no food.

## AUTHOR CONTRIBUTIONS

**Nicole N. Nakata:** Conceptualization (lead); data curation (lead); formal analysis (lead); funding acquisition (equal); investigation (lead); methodology (lead); project administration (lead); resources (supporting); writing – original draft (lead). **Richard B. Emlet:** Conceptualization (supporting); funding acquisition (equal); resources (lead); writing – review and editing (lead).

## FUNDING INFORMATION

William R. Sistrom Memorial Fellowship of the University of Oregon, Charles Lambert Memorial Fellowship of the Friday Harbor Laboratories (partial funding), and NSF grant OCE 1950520 to R. Emlet and M. Watts.

## CONFLICT OF INTEREST STATEMENT

These authors declare no conflict of interest.

## Supporting information


Table S1
Table S2
Table S3
Table S4

## Data Availability

The data that support the findings of this study are available in BOLD at boldsystems.org, or GenBank at https://www.ncbi.nlm.nih.gov, under the reference numbers listed in Table 2.
