# Supplementary material for: Having cake and eating too: The benefits of an intermediate larval form in a brittle star Amphiodia sp. opaque (Ophiuroidea)
Source: Ecol Evol. 2023 Jul 17;13(7):e10298. doi: 10.1002/ece3.10298 (PMC10352130; doi:10.1002/ece3.10298)
Supplement: Supplementary file 1 — Table S1 Table S2 Table S3 Table S4 [file ECE3-13-e10298-s001.docx]

## SUPPLEMENTARY TABLES

Table S1. Pairwise comparisons for percent metamorphosis. P-values adjusted with the Holm method. Significant values are marked with and asterisk (*).

| Comparison | Z | P | P adjusted |
| --- | --- | --- | --- |
|  | | | |
| Food 2020 – food 2021 | 2.97 | 0.003 | 0.015 * |
| Food 2020 – no food 2020 | 5.35 | < 0.001 | < 0.001 * |
| Food 2021 – no food 2020 | 1.75 | 0.080 | 0.160 |
| Food 2020 – no food 2021 | 2.12 | 0.034 | 0.102 |
| Food 2021 – no food 2021 | -0.76 | 0.444 | 0.444 |
| No food 2020 – no food 2021 | -2.60 | 0.009 | 0.038 * |

Table S2. Pairwise comparisons from Dunn’s test for juvenile aboral surface area according to treatment and year: 2020 (20), 2021 (21).

| Comparison | Z | P.unadj | P.adj |
| --- | --- | --- | --- |
|  | | | |
| Food 20 – food 21 | -0.558 | 0.577 | 1.000 |
| Food 20 – no food 20 | 3.641 | 0.000 | 0.002 * |
| Food 21 – no food 20 | 3.814 | 0.000 | 0.001 * |
| Food 21 – no food 21 | 2.765 | 0.006 | 0.028 * |
| Food 21 – no food 21 | 3.006 | 0.003 | 0.019 * |
| No food 20 – no food 21 | -1.071 | 0.284 | 1.000 |
| Food 20 – wild 19 | 2.766 | 0.006 | 0.034 * |
| Food 21 – wild 19 | 3.007 | 0.003 | 0.021 * |
| No food 20 – wild 19 | -0.520 | 0.603 | 1.000 |
| No food 21 – wild 19 | 0.443 | 0.657 | 0.657 |

Table S3. Akaike’s (AIC) and Bayesian Information Criteria (BIC) values for generalized linear models of juvenile aboral surface area (juv.size) in response to planktonic duration (p.d.). Models with the lowest AIC or BIC values are bolded.

| Model | | | df | AIC | ΔAIC | BIC | ΔBIC |
| --- | --- | --- | --- | --- | --- | --- | --- |
|  |  |  | | |  |  |  |
| Juv. size ~ p.d. | | | 3 | -244.4 | 43.9 | 587.2 | 37.2 |
| **Juv. size ~ p.d. + treatment** | | | **4** | **-288.3** | **-** | **550.0** | **-** |
| Juv. size ~ p.d. + year | | | 4 | -286.3 | 2 | 570.7 | 20.7 |
| Juv. size ~ p.d. + treatment + year | | | 5 | -268.4 | 19.9 | 553.3 | 3.3 |

Table S4. Akaike’s (AIC) and Bayesian Information Criteria (BIC) value for generalized linear models of juvenile starvation time (days) based on juvenile aboral surface area. AIC weights are *w*. Models with the lowest AIC or BIC values are bolded.

| Model | df | AIC | ΔAIC | BIC | ΔBIC |
| --- | --- | --- | --- | --- | --- |
|  |  |  |  |  |  |
| Starve time ~ juv. size | 3 | 2120.9 | 459.2 | 2125.4 | 453.2 |
| Starve time ~ juv. size + treatment | 5 | 1978.7 | 317.0 | 1986.2 | 314.0 |
| Starve time ~ juv. size + year | 5 | 1703.7 | 42.1 | 1711.2 | 39.1 |
| Starve time ~ juv. size + treatment + year | 6 | 1686.1 | 24.5 | 1695.1 | **23.0** |
| **Starve time ~ juv. size + treatment * year** | **7** | **1661.7** | **-** | **1672.1** | - |
